# Genetic architecture differences between pediatric and adult-onset inflammatory bowel diseases in the Polish population

**DOI:** 10.1038/srep39831

**Published:** 2016-12-23

**Authors:** Jerzy Ostrowski, Agnieszka Paziewska, Izabella Lazowska, Filip Ambrozkiewicz, Krzysztof Goryca, Maria Kulecka, Tomasz Rawa, Jakub Karczmarski, Michalina Dabrowska, Natalia Zeber-Lubecka, Roman Tomecki, Anna Kluska, Aneta Balabas, Magdalena Piatkowska, Katarzyna Paczkowska, Jaroslaw Kierkus, Piotr Socha, Michal Lodyga, Grazyna Rydzewska, Maria Klopocka, Grazyna Mierzwa, Barbara Iwanczak, Elzbieta Krzesiek, Katarzyna Bak-Drabik, Jaroslaw Walkowiak, Beata Klincewicz, Piotr Radwan, Urszula Grzybowska-Chlebowczyk, Piotr Landowski, Agnieszka Jankowska, Bartosz Korczowski, Teresa Starzynska, Piotr Albrecht, Michal Mikula

**Affiliations:** 1 Department of Gastroenterology and Hepatology, Medical Center for Postgraduate Education, Warsaw 01-813, Poland; 2 Department of Genetics, Cancer Center-Institute, Warsaw 02-781, Poland; 3 Department of Pediatric Gastroenterology and Nutrition, Medical University of Warsaw, Warsaw 02-091, Poland; 4 Department of Gastroenterology, Hepatology and Feeding Disorders, Children’s Memorial Health Institute, Warsaw 04-730, Poland; 5 Department of Internal Medicine and Gastroenterology with IBD Subdivision, Central Clinical Hospital of the Ministry of the Interior, Warsaw 02-507, Poland; 6 Faculty of Health Sciences, Jan Kochanowski University, Kielce 25-369, Poland; 7 Vascular Diseases and Internal Medicine, Nicolaus Copernicus University in Torun, Collegium Medicum, Bydgoszcz 85-067, Poland; 8 Department of Pediatrics, Gastroenterology and Nutrition, Wroclaw Medical University, Wroclaw 50-367, Poland; 9 Department of Pediatrics, School of Medicine with the Division of Dentistry in Zabrze, Medical University of Silesia, Katowice 40-752 Poland; 10 Department of Pediatric Gastroenterology & Metabolic Diseases, Poznan University of Medical Sciences, Poznan 61-701, Poland; 11 Department of Gastroenterology, Medical University of Lublin, Lublin 20-059, Poland; 12 Department of Pediatrics, School of Medicine in Katowice, Medical University of Silesia, Katowice 40-752, Poland; 13 Department of Pediatrics, Pediatric Gastroenterology, Hepatology and Nutrition, Medical University of Gdansk, Gdansk 80-210, Poland; 14 Medical College, University of Rzeszow, Rzeszow 35-959, Poland; 15 Department of Gastroenterology, Pomeranian Medical University, Szczecin 70-204, Poland

## Abstract

Most inflammatory bowel diseases (IBDs) are classic complex disorders represented by common alleles. Here we aimed to define the genetic architecture of pediatric and adult-onset IBDs for the Polish population. A total of 1495 patients were recruited, including 761 patients with Crohn’s disease (CD; 424 pediatric), 734 patients with ulcerative colitis (UC; 390 pediatric), and 934 healthy controls. Allelotyping employed a pooled-DNA genome-wide association study (GWAS) and was validated by individual genotyping. Whole exome sequencing (WES) was performed on 44 IBD patients diagnosed before 6 years of age, 45 patients diagnosed after 40 years of age, and 18 healthy controls. Altogether, out of 88 selected SNPs, 31 SNPs were replicated for association with IBD. A novel *BRD2* (rs1049526) association reached significance of *P* = 5.2 × 10^−11^ and odds ratio (OR) = 2.43. Twenty SNPs were shared between pediatric and adult patients; 1 and 7 were unique to adult-onset and pediatric-onset IBD, respectively. WES identified numerous rare and potentially deleterious variants in IBD-associated or innate immunity-associated genes. Deleterious alleles in both groups were over-represented among rare variants in affected children. Our GWAS revealed differences in the polygenic architecture of pediatric- and adult-onset IBD. A significant accumulation of rare and deleterious variants in affected children suggests a contribution by yet unexplained genetic components.

Inflammatory bowel diseases (IBDs) are chronic disorders with disease onset ranging from early childhood to beyond the sixth decade of life. Childhood-onset IBD represents 10–25% of all IBD cases^1^,^2^,^3^,^4^. Most IBDs, including Crohn’s disease (CD) and ulcerative colitis (UC), are classic complex disorders^5^ underlined by genetic variance which may affect the patient’s defense and adaptive mechanisms to environmental factors. Their genetic load is represented by common alleles that, in response to intestinal microbiota, underlie multiple intestinal immunopathological processes^3^,^4^. However, a minority of patients with adult-onset IBD and an unknown fraction of pediatric patients, especially those with very early-onset (VEO) disease (<6 years old), may represent monogenic disorders with an IBD-like presentation. These monogenic defects have been found to also alter intestinal immune homeostasis, with defective processing of intracellular bacteria, autophagy, and innate immunity in CD and disruption of the epithelial barrier along with the epithelial response in UC^2^,^6^.

Genome-wide association studies (GWASs) provide information on common variants associated with disease susceptibility. The largest genetic association study for IBD included >75,000 patients and controls which uncovered 163 susceptibility loci; 110 of them were shared between CD and UC, whereas 30 and 23 were unique to CD and UC, respectively. Each locus contained an average of five genes^7^. All but a few loci (e.g., NOD2 and IL23R) exhibit a rather tiny size effect (odds ratio (OR) <1.3)^8^ and contribute individually to only a small proportion of the expected heritability in IBDs. Multiple known alleles in nearly 200 loci associated with IBD explain only 13.6% and 7.5% of the overall disease variance of CD and UC risk, respectively^7^.

Pediatric IBDs are typically characterized by a more extensive disease course, a change in disease location over time, and a more frequent positive family history of IBD. In contrast, patients diagnosed between the ages of 20 and 30 years have a relatively less variable phenotype, and those diagnosed after the age of 60 years often have a mild disease severity^1^,^9^,^10^,^11^. Although the IBD location, progression, and response to therapy depend on the age of onset, multiple determinants of the early age of IBD onset remain largely unknown.

The previously reported pediatric GWASs identified loci that mostly replicated adult-onset IBD studies^12^. Although underpowered, GWASs carried out exclusively in pediatric patients uncovered 23 of 32 loci previously found in adult-onset CD and 8 of 17 loci previously found in adult-onset UC. Another report^14^ based on SNPs associated with pediatric-onset IBD suggested a role of *NOD2, TNFSF15, POU5F1*, and HLA-DRB1*501 in pediatric-onset CD and *LAMB1* in pediatric-onset UC. However, few pediatric-onset disease-associated loci have been described, including *20q13, 21q22,* and *16p11*^15^,^16^. As in GWASs of adult-onset IBD, most loci ascertained by pediatric GWASs have a small effect size. Nevertheless, genetic load has been speculated to contribute to IBD etiology and that the phenotype across ages is greater in pediatric-onset than in adult-onset IBD^2^,^17^.

To better define risk variants across pediatric- and adult-onset IBDs and identify additional susceptibility loci in the Polish population, the genetic architecture of IBDs was analyzed simultaneously in pediatric and adult cohorts using a pooled-DNA sample-based GWAS to screen for IBD associations. The GWAS findings were further validated using individual DNA samples from enlarged patient cohorts and TaqMan SNP Genotyping Assays. In addition, we searched for rare genetic variants in select sub-groups of patients with VEO and adult-onset IBD using whole exome sequencing (WES).

## Materials and Methods

### Ethics statement

All procedures performed in studies involving human participants were in accordance with the ethical standards of the institutional and/or national research committee and with the 1964 Helsinki declaration and its later amendments or comparable ethical standards. The study was approved by the Ethics Committee of the Medical Center for Postgraduate Education, Warsaw, Poland. Informed consent was obtained from all individual participants included in the study.

### Subjects

Between 2010 and 2014, a total of 1495 patients without an IBD family history were recruited at 17 Gastroenterology Departments at different Polish hospitals; 761 were diagnosed with CD and 734 were diagnosed with UC. IBDs were diagnosed in children and adolescents according to the Porto criteria modified in accordance with the recommendations of the European Crohn’s and Colitis Organization (ECCO), and in adults according to ECCO guidelines.

The GWAS cohort comprised 594 patients diagnosed with CD (356 before 17 years of age), 571 patients diagnosed with UC (311 before 17 years of age), and 724 healthy controls. Larger cohorts of cases and controls were enrolled in a replication study, including 761 patients diagnosed with CD (424 before 17 years of age), 734 patients diagnosed with UC (390 before 17 years of age), and 934 healthy controls. Sample sizes and the age distribution of each group are shown in Supplementary Table 1.

See the Supplementary Materials and Methods for detailed description of GWAS, individual genotyping and WES, analysis of mutation, and statistical analysis.

## Results

For the association screening, we conducted a cost-effective GWAS. DNA samples from individual patients that passed quality control were equimolarly combined according to patient diagnosis, sex, and age at disease onset to obtain 49 and 30 DNA pools representing 1165 IBD patients and 724 healthy controls, respectively. Of these DNA pools, 9 and 16 represented adult-onset UC (diagnosed ≥17 years of age) and pediatric-onset UC (diagnosed <17 years of age), respectively, and 10 and 14 represented adult- and pediatric-onset CD, respectively. The susceptibility loci for IBD in the Polish population were selected by comparing genome-wide genotypes between healthy controls and patients with IBD (both UC and CD), with UC only, and with CD only. A total of nine separate comparisons were performed for the following groups: adult and pediatric-onset, adult-onset only, and pediatric-onset only. Selected SNPs identified by the GWAS were validated using individual TaqMan SNP genotyping assays for the enlarged cohorts of 761 CD patients, 734 UC patients, and 934 healthy controls.

### Association screening and validation assay

A method of selecting loci for validation is crucial for the success of GWASs. In this study, we selected SNPs using three distinct criteria. First, we automatically uncovered 24 SNPs from 20 distinct loci associated at *P* < 5 × 10^−8^ (a standard genome-wide significance threshold) in at least one comparison (Supplementary Table 2). Although about half of SNPs selected for chip construction have a MAF >5% in Caucasians, among the 24 SNPs with the highest level of association, 6 were low-frequency variants (MAF = 0.5–5%) and 9 were rare variants (MAF <0.5%). Consequently, out of 20 SNPs subjected to validation (four were not validated for technical reasons), a minor allele was detected for only 7. In line with this observation, among 124 SNPs associated at a *P*-value between 5 × 10^−8^ and 10^−6^, 9 and 94 SNPs were low-frequency and rare variants, respectively, according to the Illumina SNP database^18^ (Supplementary Table 2). Because rare and low-frequency variants require a very large cohort to achieve a significant association, most of those SNPs were likely false-positive. In the 7 validated findings, 6 SNPs reached the level of significance after multiple test correction, and one SNP was at the nominal level of significance (*P* < 0.05) (Supplementary Tables 3 and 4). Of these. 5 SNPs were located in close proximity to several other alleles associated in the GWAS with a disease at the same or lower level of significance.

Assuming that an “index SNP” at a given locus is usually not independent of neighboring SNPs, we next focused on loci forming blocks of at least 10 SNPs that remained in strong allele linkage disequilibrium; the distance between two SNPs in the block was less than 30 kb and each SNP associated with a disease was at *P* < 0.005. Among 72 selected blocks, including 5 loci from the first selection, 55 consisted of at least 10 SNPs associating at *P* < 0.001 (Supplementary Table 4). Among 67 “index SNPs” from blocks of the second selection, the TaqMan SNP genotyping assays validated association of 62 SNPs at least at the nominal level of significance and OR > 1.2 OR < 0.83 in at least one of nine comparisons, and multiple test correction reduced the number of significant associations to 20 SNPs (Table 1 and Supplementary Table 4). All of the SNPs had the same direction of effect as observed in the GWAS. Altogether, selection of “index SNPs” according to allele linkage disequilibrium reduced the number of false-positive genome-wide associations.

Finally, we selected 14 “index” SNPs from “incomplete” blocks (e.g., composed of a smaller number of SNPs and associating at a *P*-value between 0.005 and 0.05) because they had associated with IBD in previous reports or represented the potentially interesting regions, such as the major histocompatibility (MHC) region. Genotyping assays validated the association of 5 SNPs at a significant level after multiple test correction and the other 9 SNPs at the nominal level of significance (Supplementary Table 4).

In summary, among 83 SNPs validated for association with the development of CD and/or UC, 31 reached the significance level after multiple test correction, and 52 SNPs were at least at the nominal level of significance. Sixty-five SNPs represented 61 susceptibility loci and 16 represented 7 HLA and 9 non-HLA genes from the MHC region (Supplementary Table 4). For 50 SNPs the minor allele was associated with an increased risk, and for 33 SNPs it had a protective effect. Thirty-four SNPs were shared between CD and UC, whereas 21 and 27 were specific for CD and UC, respectively. All of the shared disease-specific SNPs had the same direction of effect in both types of IBD.

**Figure 1:**
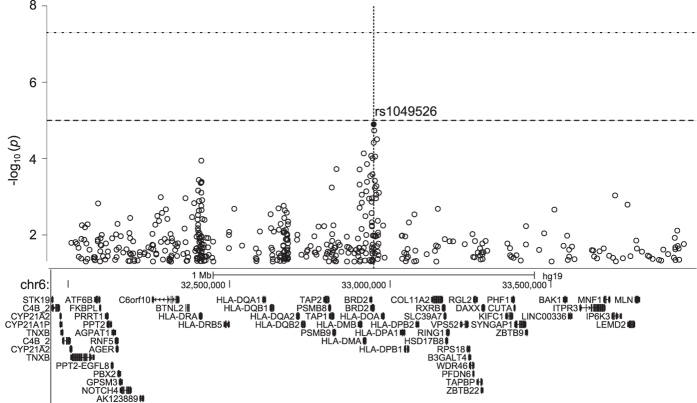
*BRD2* rs1049526-centered Manhattan plot of genome-wide associations for Crohn’s disease compared to healthy controls. The *x*-axis represents a 2 Mb window with the physical order of the genes. The dashed and dotted horizontal lines indicate the significance threshold of *P* = 1 × 10^−5^ and *P* = 5 × 10^−8^, respectively.

A large number of previously identified loci exhibited a relatively small effect size with an OR < 1.3. In line with this, the genotyping assays revealed a small effect size (1.5 > OR > 1.2 or 0.63 < OR < 0.83) of the association for 53 SNPs, a moderate effect size (2.0 > OR > 1.5 or 0.5 < OR < 0.63) of the association for 22 SNPs and a strong effect size with OR > 2.0 or OR < 0.5 for only eight SNPs. Among the top findings, three SNPs in the NOD2 locus (rs13333062, OR 2.35, *P* = 3.5 × 10^−22^, intergenic location; rs6596, OR 3.74, *P* = 1.7 × 10^−54^, SNX20 coding location; rs2076756, OR 2.52, *P* = 8.1 × 10^−36^, NOD2 intron location) were associated with an increased risk of CD and one SNP in the IL23R locus (rs11209026, OR 0.24, *P* = 1.9 × 10^−6^, coding location) was associated with a decreased risk of both UC in adult patients and CD in the two age groups. A new strong association signal found in the MHC region (SNP rs1049526 located in the 3′UTR of *BRD2*, minimum *P* = 5.2 × 10^−11^, OR 2.22–2.70) was associated with an increased risk of IBD, independent of IBD onset (Fig. 1). None of the 15 additional SNPs from this region that were associated with increased or decreased risk reached the effect size of *BRD2* rs1049526.

We then assessed the differences in genetic architecture between pediatric- and adult-onset disease (Table 1). Eleven SNPs were shared between pediatric and adult patients; 5, 5, and 1 were unique to IBD, CD, and UC, respectively. An additional 9 SNPs shared only partly different IBD subtypes between the two patient age groups. Of the remaining associations, one SNPs was unique to the adult-onset population in UC group and 8 SNPs were unique to the pediatric-onset population with 3, 3, and 2 unique to IBD, CD, and UC, respectively. Eleven additional SNPs at the nominal level of significance were shared (completely or partly) between the two patient age groups while 20 and 21 nominally significant SNPs were unique to the adult- or pediatric-onset population, respectively (Supplementary Table 4).

### Exome sequencing

GWASs have limited utility in identifying low frequency and rare variants with greater penetrance and true causal genetic variants associated with VEO IBD^19^. To further search for potential differences in the genetic architecture of VEO and adult-onset IBD, we performed WES analysis for 21 and 22 children diagnosed with CD and UC, respectively, at less than 6 years of age (age range 1–5; median −3) and 23 and 22 patients diagnosed with CD and UC, respectively, after 40 years of age (age range 41–60; median −48.5). In addition, WES was conducted in 18 healthy individuals. Before filtering, sequencing resulted in 150,314 variants discovered: 131,884 SNVs and 18,722 Indels. Each sample had an average 447 Indels and 17,790 SNVs, 640 of which were private variants (sequencing details in Supplementary Table 5).

We identified a total of 2615 rare, homozygous and heterozygous variants (SNPs and Indels), defined by a MAF <2% in the 1 kGP, European-American MAF in the NHLBI Exome Sequencing Project and ExaC, or as novel variants in coding regions of genes selected from an extended list of genes associated with CD or UC^20^,^21^. Among these, 1255 variants were categorized as deleterious (Supplementary Table 6) according to the criteria described in the Supplementary Materials and Methods. Analyzing deleterious variant accumulation in genes defined a priori as associated with IBD risk (list provided by Jostins *et al*.^7^), we found them to be over-represented among rare variants in VEO IBD patients when compared to both healthy controls and adult IBD patients (Table 2). In contrast, no differences were found in the accumulation of these deleterious variants between adult patients and healthy controls.

Of 2072 rare and novel homozygous and heterozygous variants discovered in coding regions of genes associated with the innate immune system in any of the sequenced exomes, 927 variants were considered deleterious (Supplementary Table 7). As presented in Table 3, these deleterious alleles were also significantly over-represented among rare variants in comparisons between VEO IBD patients and healthy controls, and between VEO and adult IBD patients. They were not over-represented in adult IBD patients compared to healthy controls. Specifically, the significant over-representation of deleterious alleles was observed in children diagnosed with CD compared to healthy controls, and in children with UC compared to affected adults (Table 3).

Of 347 deleterious homozygous variants present in affected children but not in affected adults or healthy controls, 28 variants were located in genes present in the extended list of genes associated with IBD^7^ (Supplementary Table 8). Six of these variants were found in more than one child (Table 4). Among the remaining 319 variants, 272 (85%) were present in only one individual. None of these variants were present in more than six individuals.

Furthermore, of 53 rare and novel non-synonymous variants (out of which 37 are possibly deleterious with CADD score on PHRED scale above 10) in genes recognized previously as being associated with monogenic IBD (in a list provided by Uhlig *et al*.^5^), two homozygote variants were found; NCF4 p.Arg8Trp in one affected adult and WAS p.Glu131Lys (rs146220228, GMAF = 0.0008) in affected child and one adult patient, but not in healthy controls. Forty-six rare variants were present in HLA genes, 16 of which were considered deleterious (Supplementary Table 9), and one homozygous highly deleterious variant (frameshift variant in HLA-DRB1, c.565_566insC) was found in an affected child. Significant over-representation of deleterious alleles among rare variants was not observed for HLA genes (Supplementary Table 10).

## Discussion

### Association studies

A majority of patients with adult-onset IBD, and possibly a significant proportion of pediatric patients with IBDs, have classic complex (multifactorial) disorders driven by multiple common genetic variants, mostly non-protein-coding SNPs, exhibiting similar small effect sizes^3^,11,^20^. However, pediatric-onset IBDs, especially VEO IBDs, may differ from adult IBDs in many aspects, including disease type, disease location, disease behavior, and gender preponderance^11^,^22^,^23^,^24^. A subset of pediatric patients with a more severe disease course is likely under a higher influence of genetic effect. To discover IBD susceptibility loci specific for pediatric-onset or adult-onset disease, we conducted association studies on adequately powered pediatric and adult cohorts simultaneously after careful selection from the Polish population by experienced gastroenterologists. Consequently, our study thus allowed direct comparisons between healthy controls, adult patients, and pediatric patients.

Association studies can focus on individually selected variants using genotyping assays or on the position of millions of DNA variants using high-throughput technologies. Generally, the greater the sample size of a GWAS, the higher the number of associations that reach the genome-wide significance threshold^25^. The largest GWAS meta-analyses of IBDs uncovered 163 susceptibility loci, with each locus containing an average of five genes^7^, whereas in a GWAS limited to hundreds of IBD patients, only a few or no associations at *P* < 5 × 10^−8^ were found. Because of the use of statistical, rather than biological, criteria^26^,^27^ GWAS may generate both false-positive and false-negative results. Therefore, findings from GWASs are commonly supported by validation and replication studies that use individual genotyping.

The independent and very reliable TaqMan SNP genotyping assays revealed that most associations selected according to the genome-wide significance threshold from our cost-effective GWAS were false-positives. In contrast, of the 72 associations which could be selected according to blocks of SNPs being in strong allele linkage disequilibrium (Supplementary Table 4, lines 3–7 and 11–77), 67 SNPs were validated by individual genotyping; of these 25 and 42 SNPs were at the corrected or the nominal level of significance, respectively. This selection criterion defined a block by a distance of less than 30 kb between each two of at least 10 SNPs associated with a disease at *P* < 5 × 10^−3^ and with an “index SNP” in the block at least at *P* < 10^−4^, but it did not take into account local probe density (number of probes in a 30,000 base pair window) and the MAF. Thus, although no extensive optimization of the number of required loci, *P*-value threshold, or window size was performed, the algorithm turned out to be surprisingly effective, possibly because of a high probes density (2,612,357) on the array used. Among verified loci there were many which haven’t passed canonical statistical criterion (examples in Supplementary Figure 1). However, it is not known whether the new associations uncovered in our study were due to DNA pooling for SNP chip hybridization in connection with a novel method for SNP selection or because they are more specific for the Polish population with a relatively higher effect size than commonly found.

Although strict Bonferroni or a similar correction for multiple comparisons is typically required to effectively minimize false positive results, this approach may also reduce the discoverable amount of the heritability by ignoring innumerable truly positive signals^27^, especially when the correction may involve tests that are correlated^25^. While the expected number of false-positive results for 83 independent tests with a significance threshold of 0.05 is close to 4, an appropriate correction for multiple comparisons would reduce this to a small fraction of 1. In our validation studies, the multiple test correction reduced the number of associations at the nominal level of significance from 83 to 31, and thus generating a large number of false-negative results and only slightly reducing the expected number of false-positive results. Furthermore, for 55 SNPs the significance threshold was reached in at least 3 out of 9 comparisons that may reduce the likelihood of a false positive for a given SNP. Therefore, we reported both uncorrected and corrected results to balance between false positives and false-negatives. To this end, an informed reader can chose between the lower false positive (Bonferroni correction) and lower false negative (no correction) option.

Altogether, we uncovered much more susceptibility loci than one would expect considering the relatively small sample size. Moreover, although all risk loci with MAF >5% and OR > 1.2 are assumed to already have been identified in IBD patients with European ancestry^8^, our study replicated only 8 known IBD loci (*NOD2, IL23R, IL10, ATG16L1, CYLD, ZMIZ1, SEMA6D,* and *CCR6*) and a few (*HLA-DRA, HLA-DQA1*) SNPs from the MHC region; all others were newly discovered associations. In line with previous reports, *NOD2* SNPs (rs13333062, rs6596, rs2076756) had the strongest effect size in both pediatric- and adult-onset CD (OR 2.3–3.7), but in contrast to other reports^28^, they did not show protective effects in UC. Among genes with SNPs exhibiting the strongest level of significance in validation genotyping were *ATG16L1, VSX2, CYLD, ADAMTS19, NOX3, TFDP1, IL23R, IL10*, and *BRD2*.

The MHC region on chromosome 6p21.3 contains more than 224 genes and is highly polymorphic. Risk variants within the MHC region are known to be associated with more than 100 different autoimmune and infectious diseases, including IBDs^2^,^29^,^30^. Although studies defining the architecture of association and causal alleles from this region are challenging, the high-density mapping of genetic variants from the MHC revealed a relatively equivalent contribution of class I and class II HLA variants to CD risk and HLA class II variation to UC risk^30^. Our study uncovered the association of 16 SNPs from the MHC region with IBDs (both risk and protective variants) (Supplementary Table 4) and one highly deleterious homozygous variant (HLA-DRB1 c.565_566insC) in an affected child. Thus, we confirmed a significant contribution of the MHC region to IBD risk and uncovered a novel signal, SNP rs1049526, located in the 3′UTR of *BRD2*, associated with an increased risk of IBD in both pediatric (OR 2.35) and adult (OR 2.66) patients.

BRD2 belongs to the bromodomain and extra-terminal domain (BET) family of chromatin adaptors that control adipogenesis, energy metabolism, and inflammation^31^. BRD2 directly regulates multiple TH17-associated cytokines, including IL17, IL21, and GMCSF^32^, and altered TH17 cells mediate autoimmune conditions, including multiple sclerosis, psoriasis, rheumatoid arthritis, and CD^32^. The mechanisms of BRD2 are in line with observations that many IBD-associated genes are involved in T-cell differentiation, specifically with the IL23 pathway (*IL23R, JAK2, STAT3, IL12B,* and *PTPN2*) involved in the maintenance of TH17 cells^28^,^33^. However, though GWASs have uncovered associations between *BRD2* SNPs and systemic sclerosis^34^, type I diabetes^35^,^36^, multiple sclerosis^37^, and rheumatoid arthritis^38^,^39^, such an association has been not reported previously for IBD. Notably, we did not confirm any association of *BRD2* rs1049526 with 458, 133, and 542 patients with primary biliary cirrhosis, primary sclerosing cholangitis, and celiac disease, respectively (data not shown). To this end, we uncovered several novel association signals, among which SNP rs1049526 in *BRD2* seems to be particularly interesting. An unresolved question that remains is whether this association is highly IBD-specific only for the Polish population or has been overlooked by other GWASs.

As reported previously, among 32 loci associated with adult-onset CD and 17 loci associated with adult-onset UC, 21 and 8 loci have been replicated in pediatric-onset CD and UC, respectively^16^. However, only a few loci (including 2q37, 10q22, 16p11, 19q13, 20q13, 21q22, and 22q12) were found to be associated with pediatric IBDs^15^,^16^,^40^. In addition, a potential role of NOD2, TNFSF15, POU5F1, HLADRB1*501, and *LAMB1* has been assumed in pediatric-onset CD and UC^14^. In this study, three *NOD2* SNPs had the same strong effect size in both pediatric- and adult-onset CD, and *IL23R* rs11209026 had a risk effect in both pediatric and adult-onset UC and in pediatric-onset CD. The pediatric-specific *ZMIZ1* rs1250550^41^ had a protective effect in pediatric-onset CD and UC as well as adult-onset CD. However, most of the SNPs uncovered in this study differentiated between pediatric- and adult-onset IBD patients.

### Exome sequencing

A diverse spectrum of rare genetic disorders with IBD-like phenotypes that result from rare causal alleles confined to exons or exon-intron boundaries^3^,^20^,^42^ can be catalogued by applying targeted exome sequencing or WES. WES interrogates the protein-coding portion of the human genome and, since its introduction, has been shown to be a powerful and cost-effective method for detecting disease variants underlying Mendelian disorders, as well as cataloguing common and rare disease-related genomic alterations^43^.

Increasing genetic burden is likely associated with an earlier age of IBD onset^20^. In support of this concept, VEO IBD patients experience distinct and more severe disease phenotypes with a more frequent positive family history^3^. Consequently, sequencing-based studies conducted in specific subsets of IBD patients (i.e., children younger than 2 years, infantile onset IBD, and familial clusters of affected individuals^44^) have discovered rare functional variants in genes implicated in the pathogenesis of both VEO (*XIAP*^45^*, FOXP3*^46^, *IL10RA, IL10RB, IL10*^47^,^48^,^49^, and *Il17REL*^42^) and adult-onset disease (*GSDMB*^21^). These monogenic defects alter intestinal immune homeostasis via several mechanisms, which were divided by Uhlig *et al*.^5^,^20^ into: (1) disruption of the epithelial barrier and the epithelial response, (2) reduced clearance of bacteria by neutrophil granulocytes and other phagocytes, and (3) altered selection and activation of T-, B-, and Treg-cells. They all account for an unknown, but probably small, fraction of VEO IBD cases.

The discovery of monogenic disorders with IBD-like phenotypes caused by highly penetrant variants and occurring more frequently in VEO patients remains within the possibilities of contemporary genetic research. On the other hand, the identification of rare, potentially pathogenic variants that do not segregate in a strict Mendelian fashion, but contribute individually to disease risk, is challenging^42^,^50^,^51^,^52^,^53^. In addition, highly penetrant variants predisposing individuals to complex disorders are likely modified by common variants with low effect size. In this study, we performed WES in 43 children diagnosed with IBD before the age of 6, 45 patients diagnosed after the age of 40, and 18 healthy adults. We identified numerous rare potentially deleterious variants in genes selected from an extended list of IBD-associated genes^7^,^20^ and genes associated with the innate immune system^54^. Both subsets of accumulated variants were over-represented in affected children, but no differences were found between adult patients and healthy controls. Our findings indicate a contribution of these variants to VEO IBD. However, because the effect size for the variants selected by WES is mainly unknown, we cannot speculate on their pathogenicity, and exact determinants of VEO IBD remain to be explained. To what extent the rare non-synonymous variants may account for “the missing heritability” of IBD is also still unknown.

Although a significantly higher aggregation of deleterious variants along large groups of genes was shown in affected children, the lack of clear pathogenic variants in genes associated previously with monogenic IBD suggests that our VEO cases represented mostly a polygenic disease. Among 53 variants in genes recognized previously as being associated with monogenic IBD, only two homozygote variants (NCF4 p.Arg8Trp and WAS p.Glu131Lys) were found in two affected adults, and homozygous WAS p.Glu131Lys was found in one adult and pediatric patient. NCF4 p.Arg8Trp is a new variant, and no clinical data is available for WAS p.Glu131Lys. *NCF4* encodes neutrophil cytosolic factor 4. This protein is a regulatory component of the superoxide-producing phagocyte NADPH-oxidase, which is crucial in host defense. Mutations in this gene cause chronic granulomatous disease^55^, in which the host’s reaction to pathogenic microbes is severely impaired. Up to 40% of patients with chronic granulomatous disease can manifest CD-like symptoms^56^, and few patients manifest symptoms of UC^57^. *WAS* encodes Wiskott-Aldrich Syndrome protein. Wiskott–Aldrich syndrome is a primary immunodeficiency disease in which up to 9% of patients exhibit an IBD-like syndrome^58^.

To this end, WES allows the cataloguing of genes enriched for rare variants, but more importantly, we need effective methods to investigate the heritability of specific functional alleles in complex disorders. The unresolved question is how to separate the individual causative effects of rare alleles with low/moderate penetrance from the effect of the polygenic burden of common variants. For the discovery of rare incompletely penetrant variants, even large studies are underpowered due to an enormous number of tests needed to establish the significance of the association^9^.

## Summary

To discover differences in the genomic architecture of IBD in Polish patients, we performed both genome-wide association screening and WES simultaneously in pediatric and adult cohorts. A pooled-DNA GWAS approach that efficiently and cost-effectively scanned for common risk alleles^59^,^60^,^61^, together with a new method for identifying SNP associations, appeared to be unexpectedly effective in identifying novel IBD susceptibility loci. Despite “index SNPs” for these loci being selected at *P*-values much higher than a standard genome-wide significance threshold, most of them were validated in individual genotyping. Our GWAS indicated differences in the polygenic architecture between pediatric- and adult-onset IBD, but a significant accumulation of missense and nonsense rare variants in affected children suggested a contribution of yet unexplained genetic components to VEO IBD. To summarize, Polish pediatric IBD patients exhibited genetically attributable risk that was visibly different from that of adult-onset patients, which may question the previous assumption^28^ of a close pathogenic relationship between pediatric- and adult-onset IBD.

## Additional Information

**Accession codes:** The datasets used in this GWAS are available in the GEO database under GSE79094. Whole-exome sequencing data (as bam files mapped to hg19 genome assembly) are available in European Nucleotide Archive under accession number PRJEB12993.

**How to cite this article**: Ostrowski, J. *et al*. Genetic architecture differences between pediatric and adult-onset inflammatory bowel diseases in the Polish population. *Sci. Rep.* **6**, 39831; doi: 10.1038/srep39831 (2016).

**Publisher's note:** Springer Nature remains neutral with regard to jurisdictional claims in published maps and institutional affiliations.

## Supplementary Material

Supplementary Information

Supplementary Tables

## Figures and Tables

**Table 1:** Summary of significant (after correction) inflammatory bowel disease (IBD), Crohn’s disease (CD), and ulcerative colitis (UC) loci validated or replicated by individual patient TaqMan genotyping.

Type	CHR	OR (CI)	SNP	Candidate genes	Location
Pediatric/Adult					
IBD**/IBD**	6	2.66 (1.93–3.66)	rs1049526	BRD2	threeUTR
IBD**/IBD**	6	1.65 (1.39–1.96)	rs2239802	HLA-DRA	intron
** IBD/**IBD	1	1.42 (1.29–1.68)	rs3024505	CNIH3	intron
IBD**/IBD**	6	1.65 (1.39–1.96)	rs2395128	HLA-DRA	downstream 500BP
** IBD/**IBD	4	1.25 (1.08–1.45)	rs13140982*	MMRN1, PYURF	intergenic
** CD/**CD	16	2.52 (2.13–2.96)	rs2076756	NOD2	intron
** CD/**CD	16	2.18 (1.81–2.64)	rs13333062	SALL1, ADCY7	intergenic
** CD/**CD	16	3.74 (3.10–4.49)	rs6596	SNX20	coding
** CD/**CD	16	0.57 (0.49–0.66)	rs3785142	CYLD	intron
** CD/**CD	7	1.39 (1.16–1.68)	rs1462278	TNS3, ADCY1	intergenic
** UC/**UC	11	1.59 (1.30–1.95)	rs56404409	OR10G8, MIR4493	intergenic
** IBD/**UC	14	1.50 (1.21–1.86)	rs4903214	VSX2	intron
IBD/**CD**	4	158 (1.23–2.04)	rs2850407	DKK2	intron
IBD/**CD**	10	0.69 (0.56–0.89)	rs1250550*	ZMIZ1	intron
CD**/IBD**	10	0.76 (0.65–0.88)	rs17098094	MIR4681, ACUL1	intergenic
CD/**IBD**	13	1.38 (1.18–1.60)	rs12429354	TFDP1	intron
CD**/IBD**	1	0.32 (0.20–0.53)	rs11209026	Il23R	coding
CD/**IBD**	6	0.51 (0.39–0.66)	rs2187668	HLA-DQA1	intron
UC/**IBD**	6	0.65 (0.53–0.79)	rs17753470	SCAF8	intergenic
CD**/UC**	4	1.84 (1.45–2.34)	rs979961	TRAM1L1, MIR1973	intergenic
** CD**/UC	11	0.71 (0.59–0.86)	rs12786216	MIR4299, MRVI1-AS1	intergenic
** CD**/UC	21	0.74 (0.63–0.87)	rs7279062	KRTAP21-3, KRTAP25–1	intergenic
Pediatric					
** IBD**	2	1.94 (1.42–2.65)	rs75975594	KIF5C	intron
** IBD**	15	1.57 (1.34–1.83)	rs501916	SEMA6D	coding
** CD**	22	1.40 (1.18–1.66)	rs5761958	MIR3199-2, MIR548J	intergenic
** CD**	2	0.74 (0.63–0.87)	rs2241880	ATG16L1	coding
** CD**	6	1.41 (1.19–1.66)	rs9459874	CCR6	intron
** UC**	21	0.73 (0.61–0.87)	rs1056930	NRIP1	threeUTR
** UC**	5	0.69 (0.58–0.83)	rs42255	ADAMTS19	intron
** **UC	2	1.34 (1.12–1.60)	rs6734351*	LINC00607	intron
Adult					
** UC**	5	1.45 (1.20–1.75)	rs10075967	SREK1IP1	threeUTR

Type: comparison in which polymorphism was significant (children/adults, bold: comparison with the lowest p-value), SNP: single nucleotide polymorphism, candidate genes: genes closest to polymorphism, location: position of polymorphism in relation to gene. CHR - chromosome, OR - Odds Ratio, CI − 95% confidence interval. OR and CI are given for the most significant (lowest p-value) comparison.

^*^SNPs significant after correction in merged young and adult groups.

**Table 2:** Fisher’s exact test for comparisons of allele frequencies between patients and healthy controls for a set of genes defined as associated with IBD risk^7^.

Comparison	*P*-value	OR	OR 95% confidence interval
HC/VEO IBD	0.01633	1.23	1.04–1.45
HC/Adult IBD	0.2222	1.11	0.94–1.32
HC/VEO UC	0.03282	1.22	1.01–1.48
HC/VEO CD	0.02665	1.23	1.02–1.48
HC/Adult UC	0.4287	1.08	0.9–1.31
HC/Adult CD	0.1607	1.14	0.95–1.38
Adult IBD/VEO IBD	0.07478	1.1	0.99–1.23
Adult CD/VEO CD	0.3574	1.07	0.92–1.25
Adult UC/VEO UC	0.1254	1.13	0.97–1.32

HC: healthy controls; CD: Crohn’s disease; UC: ulcerative colitis; VEO: very early-onset; IBD: inflammatory bowel disease; OR: Odds ratio.

**Table 3:** Fisher’s exact test for comparisons of allele frequencies between patients and healthy controls for a set of genes associated with the innate immune system.

Comparison	*P*-value	OR	OR 95% confidence interval
HC/VEO IBD	0.01061	1.27	1.05–1.54
HC/Adult IBD	0.7369	1.03	0.85–1.26
HC/VEO UC	0.08641	1.2	0.97–1.48
HC/VEO CD	0.004451	1.34	1.09–1.66
HC/Adult UC	0.5968	0.94	0.77–1.17
HC/Adult CD	0.2206	1.14	0.92–1.42
Adult IBD/VEO IBD	0.0007325	1.23	1.09–1.39
Adult CD/VEO CD	0.06776	1.17	0.99–1.4
Adult UC/VEO UC	0.006933	1.27	1.07–1.51

HC: healthy controls; CD: Crohn’s disease; UC: ulcerative colitis; VEO: very early-onset; IBD: inflammatory bowel disease; OR: Odds ratio

**Table 4:** Homozygous deleterious variants in genes associated with inflammatory bowel disease that were present in more than one child and not present in adults or healthy controls as homozygotes.

Chr	Pos	rs	type	Gene	HGV	Number
1	1372400	.	missense	VWA1	ENSP00000417185.1:p.Phe56Cys	6
19	48997185	.	missense	LMTK3	ENSP00000270238.3:p.Glu1315Asp	4
15	41313211	.	frameshift	INO80	ENST00000361937.3:c.3160delG	3
2	27804634	.	missense	C2orf16	ENSP00000386190.2:p.Arg1732Lys	2
3	48667556	.	missense	SLC26A6	ENSP00000378920.2:p.Lys454Thr	2
16	50763778	rs2066847	frameshift	NOD2	ENST00000300589.2:c.3019dupC	2

Genomic coordinates are given for GRCh37 assembly. Chr: chromosome; Pos: position; rs: id in dbSNP if available; type: mutation type; HGV: Human Genome Variation annotation; number: number of homozygous VEO IBD patients.
