# Supplementary material for: Genetic architecture differences between pediatric and adult-onset inflammatory bowel diseases in the Polish population
Source: Sci Rep. 2016 Dec 23;6:39831. doi: 10.1038/srep39831 (PMC5180213; doi:10.1038/srep39831)
Supplement: Supplementary Information [file srep39831-s1.pdf]

Supplementary information

Genetic architecture differences between pediatric and adult-onset inflammatory bowel diseases in the Polish population

Jerzy Ostrowski[1,2], Agnieszka Paziewska[1], Izabella Lazowska[3], Filip Ambrozkiewicz[1], Krzysztof Goryca[2], Maria Kulecka[1], Tomasz Rawa[1], Jakub Karczmarski[2], Michalina Dabrowska[2], Natalia Zeber-Lubecka[1], Roman Tomecki[1], Anna Kluska[2], Aneta Balabas[2], Magdalena Piatkowska[2],Katarzyna Paczkowska[2], Jaroslaw Kierkus[4], Piotr Socha[4], Michal Lodyga[5], Grazyna Rydzewska[5,6], Maria Klopocka[7], Grazyna Mierzwa[7], Barbara Iwanczak[8], Elzbieta Krzesiek[8], Katarzyna Bak-Drabik[9], Jaroslaw Walkowiak[10], Beata Klincewicz[10], Piotr Radwan[11], Urszula Grzybowska-Chlebowczyk[12], Piotr Landowski[13], Agnieszka Jankowska[13], Bartosz Korczowski[14], Teresa Starzynska[15], Piotr Albrecht[3], Michal Mikula[2]

[1]Department of Gastroenterology and Hepatology, Medical Center for Postgraduate Education, Warsaw 01-813, Poland; [2]Department of Genetics, Cancer Center-Institute, Warsaw 02-781, Poland; [3]Department of Pediatric Gastroenterology and Nutrition, Medical University of Warsaw, Warsaw 02-091, Poland; [4]Department of Gastroenterology, Hepatology and Feeding Disorders, Children's Memorial Health Institute, Warsaw 04-730, Poland; [5]Department of Internal Medicine and Gastroenterology with IBD Subdivision, Central Clinical Hospital of the Ministry of the Interior, Warsaw 02-507, Poland; [6] Faculty of Health Sciences, Jan Kochanowski University, Kielce 25-369, Poland; [7]Vascular Diseases and Internal Medicine, Nicolaus Copernicus University in Torun, Collegium Medicum, Bydgoszcz 85-067, Poland; [8]Department of Pediatrics, Gastroenterology and Nutrition, Wroclaw Medical University, Wroclaw 50-367, Poland; [9]Department of Pediatrics, School of Medicine with the Division of Dentistry in Zabrze, Medical University of Silesia, Katowice 40-752 Poland; [10]Department of Pediatric

Gastroenterology & Metabolic Diseases, Poznan University of Medical Sciences, Poznan 61-701, Poland; [11]Department of Gastroenterology, Medical University of Lublin, Lublin 20-059, Poland; [12]Department of Pediatrics, School of Medicine in Katowice, Medical University of Silesia, Katowice 40-752, Poland; [13]Department of Pediatrics, Pediatric Gastroenterology, Hepatology and Nutrition, Medical University of Gdansk, Gdansk 80-210, Poland; [14]Medical College, University of Rzeszow, Rzeszow 35-959, Poland; [15]Department of Gastroenterology, Pomeranian Medical University, Szczecin 70-204, Poland

Correspondence: Jerzy Ostrowski, MD, PhD; Cancer Center-Institute, Roentgena 5, 02-781 Warsaw, Poland, Tel: +48 225462575, E-mail: jostrow@warman.com.pl

Supplementary Figure 1. Manhattan plots for loci found to be significant in comparison of all IBD patients vs controls at the GWAS stage and positively verified by Taqman genotyping.

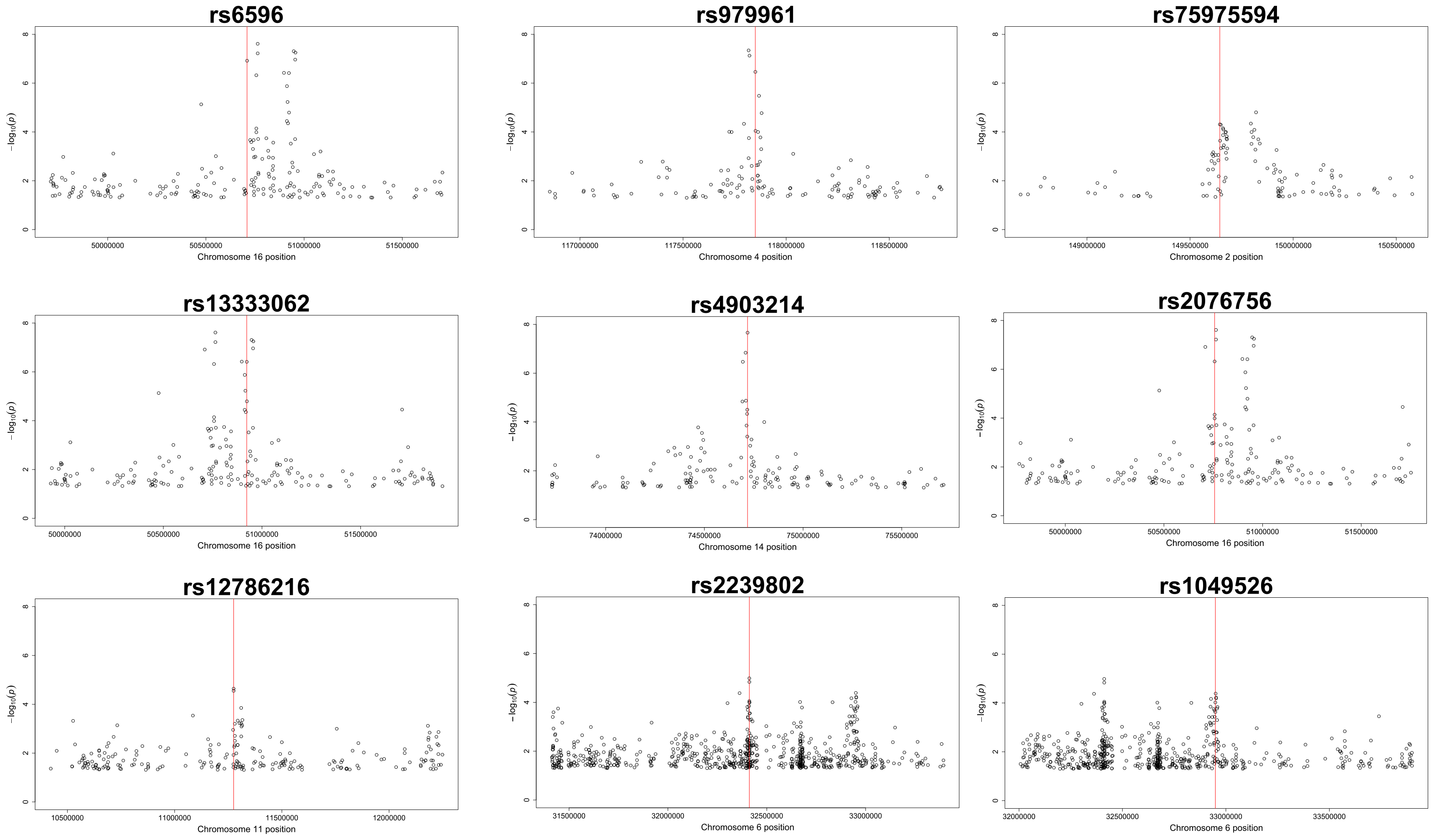

Supplementary Materials and Methods

Allelotyping GWAS

A pooled-DNA sample-based GWAS was performed as described previously [1]. Genomic DNA was extracted from whole blood treated with EDTA using a QIAamp DNA Mini Kit. DNA concentrations were measured using a Quant-iT^{TM} PicoGreen dsDNA Kit (Invitrogen, United Kingdom). DNA integrity was verified by 1% agarose gel analysis.

DNA samples that passed quality control tests were grouped at equimolar concentrations by patient age at diagnosis, sex, and IBD diagnosis in order to obtain pools consisting of 23-24 DNA samples. Pooled DNA samples were adjusted to a final concentration of 50 ng/ml in Tris-EDTA buffer (pH 8). A total of 25, 24, and 30 DNA pools were prepared for CD, UC, and control samples, respectively. DNA pools were assayed independently on Illumina Human Omni2.5-Exome BeadChips by the external AROS Applied Biotechnology A/S (Aarhus, Denmark) service. The datasets used in this GWAS are available in the GEO database under GSE79094.

Statistical analysis

For each SNP on each microarray in the GWAS, the relative allele signal (RAS) was calculated as described previously [1]. The RAS was used as an approximation of the allele ratio. Due to a lack of call-rate statistics for pooled samples, quality was assessed by visual inspection of the first two principal components. Principal component analysis (PCA) was performed for subsets of 250,000 probes due to memory constraints. Six control pools and three UC pools were removed as outliers (Supplementary Figure 1). No probe filtering was performed. The Student's t-test (Welch variant) was used to compare allele ratios between groups. Distribution

assumptions were verified by visual inspection of the *t*-statistic QQ plot (Supplementary Figure 2). Genomic inflation factor λ was also computed. It is defined as the ratio of the median observed and expected test statistics (Supplementary Materials and Methods Table 1). To obtain informative λ values for the Student's t-test, we modified the formula by taking the absolute values of the observed and expected statistics. *P*-values were corrected for multiple hypothesis testing using the Benjamin-Hochberg algorithm to control the false discovery rate (FDR) [2]. Manhattan plotting was performed using the qqman R package [3]. All computations were performed in the R environment [4].

Individual genotyping

To validate the findings of the GWAS and replicate the SNP typing study, individual patients and controls were genotyped using TaqMan SNP Genotyping Assays (Life Technologies, USA) with TaqMan Universal Master Mix II and a 7900HT Real-Time PCR system (Life Technologies, USA). Associations were examined by the Fisher-exact test implemented in R (version 3.1.1). The ORs and 95% confidence intervals (CIs) were estimated by normal approximation using the EpiTools R package [5]. Significance threshold was set at 6.67E-4 (0.05/75), because 88 assessed SNPs represented 75 LD blocks.

Exome sequencing

A human exome sequencing library was prepared using the Ion AmpliSeq™ Exome Kit (Thermo Fisher) according to the manufacturer's protocol. Briefly, 100 ng of genomic DNA was subjected to multiplex amplification with 2x Exome Primer Pool. Next, primers were digested and adapters ligated to the amplicons. The samples were then purified using Agencourt AMPure XP beads (Beckman Coulter) and stored at -20°C for further processing. The concentration of

each library was determined using a Qubit fluorometer (Thermo Fisher) and DNA fragment length assessed using High Sensitivity DNA Analysis Kits on a Bioanalyzer 2100 (Agilent). Each library was diluted to ~100 pM prior to template preparation. Up to three barcoded libraries were subjected to automated template preparation with an Ion PI IC 200 Kit on the Ion Chef Instrument, which performs emulsion PCR on Ion Sphere Particles, followed by particle recovery and template loading on a PI chip. Samples were sequenced in an Ion Proton instrument on a PI chip using the sequencing reagents provided as part of the Ion PI IC 200 Kit according to the manufacturer's instructions. Whole-exome sequencing data (as bam files mapped to hg19 genome assembly) are available in European Nucleotide Archive under accession number PRJEB12993

Read mapping and variant calling

Raw reads were processed by the Torrent Suite analysis pipeline and mapped to human genome assembly hg19 by TMAP [6]. Variant calls were made by Torrent Variant Caller [7] with the parameters detailed in Supplementary Materials and Methods Table 2. Variant calls were filtered by the variant-filter script [8] with default parameters, which was modified to filter small insertions and deletions as well as single nucleotide variants. Variants were further analyzed if they passed the filter for at least one individual. If multiple alleles were reported in one position, they were separated by vcflib script vcfbreakmulti [9]. Variants were annotated by Variant Effect Predictor [10] release 83.

Deleterious variants

A variant was deemed deleterious if it met three criteria. First, its impact, as determined by Variant Effect Predictor, was not 'low'. Second, it was not previously reported as benign or

likely benign in ClinVar. Finally, its CADD [11] score was at least 10 on the PHRED scale. The last criterion was based on cut-off provided by Kelsen *et al* [12].

We investigated rare (MAF < 2%) deleterious variants present as homozygotes only in children (i.e., present in neither affected adults nor healthy controls). This allele frequency is based on frequency of rare, deleterious variant rs2066847 associated with IBD in European population. We took into special consideration variants present in histocompatibility complex (HLA) genes, variants in genes previously associated with monogenic IBD according to the list provided by Uhlig et al. [13] (50 genes), and variants in genes previously associated with IBD according to the list provided by Jostins et al. [14] (1715 genes).

Over-representation of deleterious alleles among rare alleles

Only variants in coding regions with a global minor allele frequency (GMAF) featured in the 1000 Genomes Project database (1kGP), European minor allele frequency (MAF) in the 1kGP, European-American MAF in the NHLBI Exome Sequencing Project, and ExaC MAF <2%, or novel variants were chosen for further analysis. Genes from HLA genes were excluded from this analysis and analyzed separately. Two subgroups of variants were analyzed: variants in genes previously associated with CD and/or UC according to the gene lists supplied by Jostins et al. [14], and variants in genes associated with the innate immune system according to Reactome [15]. The over-representation of deleterious alleles was determined by Fisher's exact test. In each individual, only one deleterious variant per gene was taken into account.

References

1. Gaj, P. *et al.* Pooled sample-based GWAS: a cost-effective alternative for identifying colorectal and prostate cancer risk variants in the Polish population. *PLoS ONE* **7,** e35307 (2012).
2. Benjamini, Y. & Hochberg, Y. Controlling the False Discovery Rate: A Practical and Powerful Approach to Multiple Testing. *Journal of the Royal Statistical Society. Series B (Methodological)* **57,** 289–300 (1995).
3. Turner, S. D. qqman: an R package for visualizing GWAS results using Q-Q and manhattan plots. *bioRxiv* 5165 (2014). doi:10.1101/005165
4. Team, R. C. *R: A language and environment for statistical computing. R Foundation for Statistical Computing, Vienna, Austria. 2013*. (ISBN 3-900051-07-0, 2014).
5. Developer, T. J. A., User, M. P. F. & User, D. W. *epitools: Epidemiology Tools*. (2012).
6. iontorrent/TS. *GitHub* Available at: https://github.com/iontorrent/TS. (Accessed: 21st February 2016)
7. TVC. Available at: http://updates.iontorrent.com/tvc_standalone/. (Accessed: 21st February 2016)
8. Kandoth, C. & Larson, D. variant-filter:A false-positive filter for variants called from massively parallel sequencing. *GitHub* Available at: https://github.com/ckandoth/variant-filter. (Accessed: 21st February 2016)
9. Garrison, E. A C++ library for parsing and manipulating VCF files. *GitHub* Available at: https://github.com/vcflib/vcflib. (Accessed: 21st February 2016)
10. McLaren, W. *et al.* Deriving the consequences of genomic variants with the Ensembl API and SNP Effect Predictor. *Bioinformatics* **26,** 2069–2070 (2010).

11. Kircher, M. *et al.* A general framework for estimating the relative pathogenicity of human genetic variants. *Nat. Genet.* **46,** 310–315 (2014).

12. Kelsen, J. R. *et al.* Exome sequencing analysis reveals variants in primary immunodeficiency genes in patients with very early onset inflammatory bowel disease. *Gastroenterology* **149,** 1415–1424 (2015).

13. Uhlig, H. H. *et al.* The diagnostic approach to monogenic very early onset inflammatory bowel disease. *Gastroenterology* **147,** 990–1007.e3 (2014).

14. Jostins, L. *et al.* Host-microbe interactions have shaped the genetic architecture of inflammatory bowel disease. *Nature* **491,** 119–124 (2012).

15. de Bono, B., Gillespie, M., Luo, F. & Gay, N. Innate Immunity Signaling. *Reactome - a curated knowledgebase of biological pathways* **24,** (2008).

Supplementary Materials and Methods Figure 1. Plot of first four principal components for: **(A, B)** full dataset **(C, D)** dataset after removal of outlier samples. CD: Crohn's disease; UC: ulcerative colitis

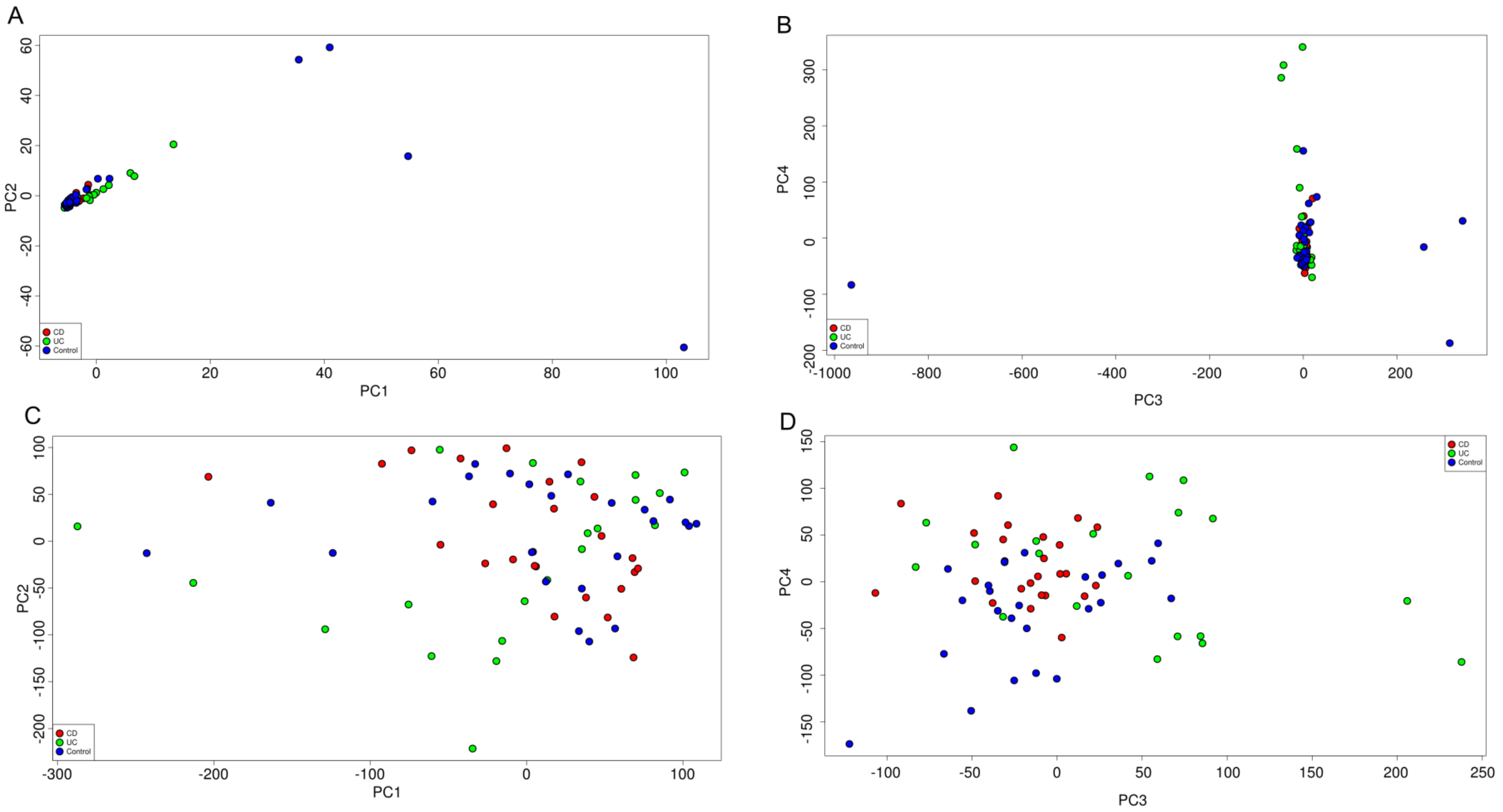

Supplementary Materials and Methods Figure 2. QQ plot of the inflammatory bowel disease GWAS. CD: Crohn's disease; UC: ulcerative colitis

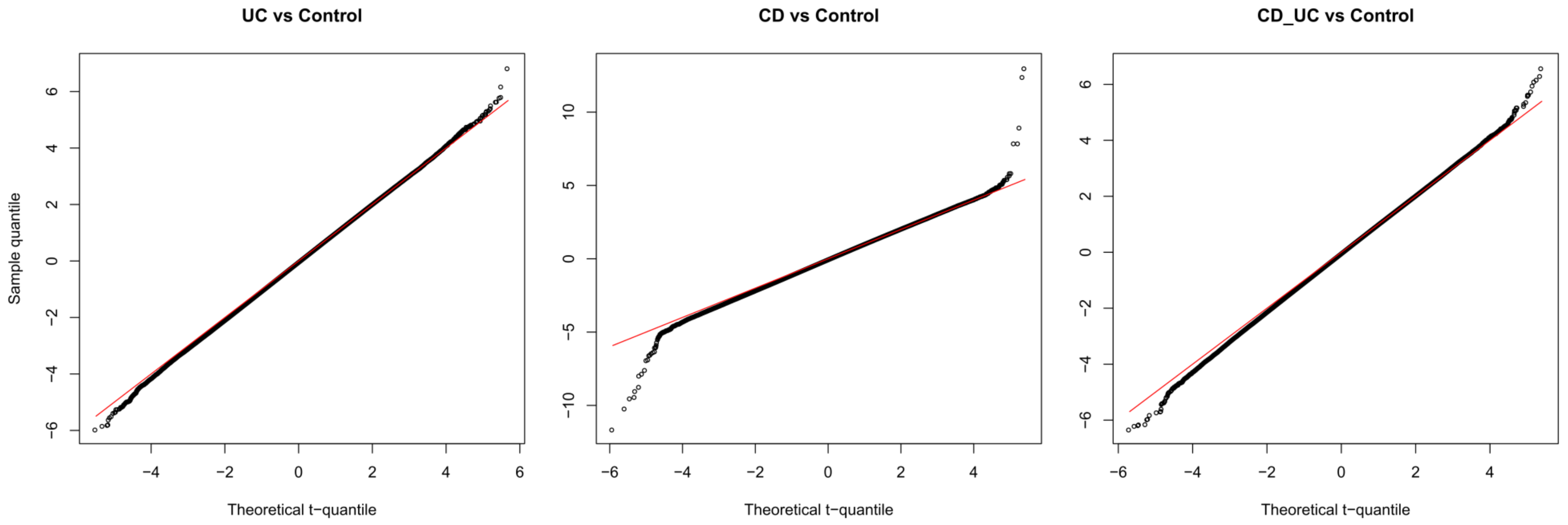

Supplementary Materials and Methods Table 1. Genomic inflation factor λ (lambda) values for a given comparison. HC: healthy controls CD: Crohn's disease; UC: ulcerative colitis; IBD inflammatory bowel disease

**grup_1**	**group_2**	**lambda**
pediatric_CD	HC	1.07
adult_CD	HC	1.11
pediatric_UC	HC	1.12
adult_UC	HC	1.08
UC	HC	1.03
CD	HC	1.06
IBD	HC	1.05
pediatric_IBD	HC	1.07
adult_IBD	HC	1.08

Supplementary Materials and Methods Table 2. Parameters for calling single nucleotide variants (SNV) and small insertions and deletions (INDEL) in Torrent Variant Caller.

Parameter	SNV	INDEL
Minimum allele frequency	0.1	0.1
Minimum quality	15	20
Minimum coverage	5	5
Minimum coverage on either strand	0	1
Maximum strand bias	0.99	0.95
Minimum relative read quality	20	20
Maximum common signal shift	0.25	0.25
Maximum reference/variant signal shift (insertions)		0.25
Maximum reference/variant signal shift (deletions)		0.25
